# *Trichomonas vaginalis* Lipophosphoglycan Exploits Binding to Galectin-1 and -3 to Modulate Epithelial Immunity*

**DOI:** 10.1074/jbc.M115.651497

**Published:** 2015-11-20

**Authors:** Raina N. Fichorova, Hidemi S. Yamamoto, Titilayo Fashemi, Evan Foley, Stanthia Ryan, Noah Beatty, Hassan Dawood, Gary R. Hayes, Guillaume St-Pierre, Sachiko Sato, Bibhuti N. Singh

**Affiliations:** From the ‡ Laboratory of Genital Tract Biology, Department of Obstetrics, Gynecology and Reproductive Biology, Brigham and Women's Hospital, Harvard Medical School, Boston, Massachusetts 02115,; the Departments of § Biochemistry and Molecular Biology and Obstetrics and Gynecology, State University of New York Upstate Medical University, Syracuse, New York 13210, and; the ¶ Laboratory of Glycobiology and Bioimaging, Research Centre for Infectious Diseases, Faculty of Medicine, Laval University, Quebec, Quebec G1V 4G2, Canada

**Keywords:** cytokine, galectin, inflammation, interleukin, parasite, Interleukin 8 (IL-8), CCL5 (RANTES), CCL-20 (MIP-3α), sexually transmitted infection, human vagina

## Abstract

Trichomoniasis is the most common non-viral sexually transmitted infection caused by the vaginotropic extracellular protozoan parasite *Trichomonas vaginalis*. The infection is recurrent, with no lasting immunity, often asymptomatic, and linked to pregnancy complications and risk of viral infection. The molecular mechanisms of immune evasion by the parasite are poorly understood. We demonstrate that galectin-1 and -3 are expressed by the human cervical and vaginal epithelial cells and act as pathogen-recognition receptors for the ceramide phosphoinositol glycan core (CPI-GC) of the dominant surface protozoan lipophosphoglycan (LPG). We used an *in vitro* model with siRNA galectin knockdown epithelial clones, recombinant galectins, clinical *Trichomonas* isolates, and mutant protozoan derivatives to dissect the function of galectin-1 and -3 in the context of *Trichomonas* infection. Galectin-1 suppressed chemokines that facilitate recruitment of phagocytes, which can eliminate extracellular protozoa (IL-8) or bridge innate to adaptive immunity (MIP-3α and RANTES (regulated on activation normal T cell expressed and secreted)). Silencing galectin-1 increased and adding exogenous galectin-1 suppressed chemokine responses to *Trichomonas* or CPI-GC/LPG. In contrast, silencing galectin-3 reduced IL-8 response to LPG. Live *Trichomonas* depleted the extracellular levels of galectin-3. Clinical isolates and mutant *Trichomonas* CPI-GC that had reduced affinity to galectin-3 but maintained affinity to galectin-1 suppressed chemokine expression. Thus via CPI-GC binding, *Trichomonas* is capable of regulating galectin bioavailability and function to the benefit of its parasitic survival. These findings suggest novel approaches to control trichomoniasis and warrant further studies of galectin-binding diversity among clinical isolates as a possible source for symptom disparity in parasitic infections.

## Introduction

Each year over 180 million people become infected with the genitourinary protozoan parasite *Trichomonas vaginalis*, which steadily accounts for over half of the sexually transmitted infections (STIs)^2^ worldwide (1) and for significant public health burdens, including low birth weight and prematurity, cervical and prostate cancer, and risk of HIV infection (2). In the United States, unlike other less common STIs, such as chlamydiasis, gonorrhea, and syphilis, trichomoniasis is not a reportable disease, and no prophylactic annual screening for *T. vaginalis* has been implemented. At the same time, the lack of symptoms in at least half of the diagnosed cases in women and even more in men suggests that in the absence of preventive screening most infections may remain undiagnosed and untreated. It is unclear how the protozoan parasite evades the immune system to allow for often silent, lasting, and recurrent infections. The parasite is best adapted to extracellular colonization of the human cervico-vaginal mucosa, and therefore, understanding the molecular mechanisms of the epithelial surface host-pathogen interactions in the human female genital tract is essential for building successful future eradication strategies.

It is known that parasitic protozoa contain a variety of complex carbohydrates on their surfaces, *e.g.* glycolipids, glycoproteins, and glycosylated phosphatidylinositol glycolipids (3). These glycoconjugates have been reported to play important roles in host cell invasion and evasion of host immune responses (4–6). *T. vaginalis* and the related bovine parasite *Tritrichomonas foetus* express lipophosphoglycans (LPG) at several million copies per parasite anchored on the protozoan surface via an inositol phosphoceramide (7, 8). We have shown that LPG assists *T. vaginalis* in adherence to the cervicovaginal epithelium (9) and in evading innate immunity by suppressing the expression of the major mucosal antimicrobial protein secretory leukocyte protease inhibitor (10, 11). The ceramide phosphoinositol glycan core (CPI-GC) of LPG also cooperates with pathogenic vaginal bacteria and with its own microflora, endobiont dsRNA *T. vaginalis* virus, to alter bacterial colonization patterns and the local immune environment in support of bacterial vaginosis, which in turn facilitates *T. vaginalis* survival (9, 12, 13). The host receptors responsible for *Trichomonas* CPI-GC signal transduction have remained elusive to date.

The *T. vaginalis* LPG and its immunocompetent CPI-GC domain contain β-galactosides and abundant poly-*N*-acetyl-lactosamine repeats (polyLacNAc) (12), which provide a molecular basis for predictable interactions with the family of galectin proteins that recognize the Galβ1–4GlcNAc disaccharide motif and polymers of that structure in the form of polyLacNAc (14–16). Furthermore, it has been reported that galectin-1 mediates the adherence of the parasite to cervical epithelial cells in an LPG-dependent manner; however, the effects of this binding on host immunity, as well as the molecular interactions of LPG and galectin-1, remained unexplored (17). We focused our studies on galectin-1 and -3 because these two galectins have shown important effects on immune cells, including phagocytes and antigen-presenting cells (18, 19), and yet their immune functions have not been studied in cervical and vaginal epithelial cells, which are the natural host for the *T. vaginalis* parasites. To our knowledge, this study is the first to identify the molecular domain on the *T. vaginalis* surface responsible for functional galectin binding that manipulates host immunity.

## Experimental Procedures

### 

#### 

##### T. vaginalis Isolates and Preparations of LPG and CPI-GC

*T. vaginalis* isolates were obtained with informed consent under IRB-approved protocol from women attending the Onondaga County Health Department STI Clinic (OC isolates) and the University Hospital Microbiology/Clinical Pathology Lab, State University of New York Upstate Medical University (UH isolates), Syracuse, NY, and the University of Rochester (the UR1 isolate) STI Clinic, Rochester, NY, as described previously(11). *T. vaginalis* wild type B7RC2 (WT) and mutants 4.12 and 2E2 were obtained from Patricia Johnson. The status of the *T. vaginalis* virus infection of each isolate was determined as described and reported (11). All *Trichomonas* isolates were cultured in modified Diamond's medium supplemented with 10% heat-inactivated horse serum (HyClone Laboratory) and iron, as reported earlier (20), harvested in late log phase (24 h) by centrifugation, washed twice with phosphate-buffered saline (PBS, pH 7.4), and suspended in methanol/chloroform (1:2) followed by LPG extraction as described previously (8). The CPI-GC core was released by mild acid hydrolysis (100 mm TFA containing 1 μg/ml DTT) and purified on a C18 Sep-Pak column. The molecular purity of the LPG and CPI-GC preparations was confirmed by mass spectrometry as described (12). In addition, the lack of endotoxin contamination of each preparation was confirmed by the EndoSafe Test System (Charles River Laboratories, Charleston, SC) based on the *Limulus* amoebocyte lysate test with sensitivity <0.05 EU/ml (12).

##### Monosaccharide Composition of LPG and CPI-GC Preparations

Monosaccharide composition of CPI-GC was determined by strong acid hydrolysis followed by high performance anion exchange chromatography. Samples (100 μg) were hydrolyzed in 4 m TFA at 125 °C for 1 h and dried under vacuum. An aliquot in water was applied to a CarboPac PA1 column (4 × 250 mm) equilibrated in 8 mm NaOH, and monosaccharides were eluted with water. Monosaccharides were detected by pulsed amperometry. In some experiments the column was equilibrated in 6 mm NaOH.

##### Recombinant Human Galectins-1 and -3 and Antibodies to Galectin-1 and -3

For the epithelial culture experiments, we used recombinant human (rh) galectin-1 and -3 generated and purified as described by Sato and co-workers (21). Recombinant galectins were purified by affinity chromatography using lactose-agarose as published previously (22–24). Purified galectin solution was passed through an ActiClean Etox (Sterogen) column to ensure that the endotoxin level was less than 10 EU/mg. For Western blot experiments, we used rh-galectin-1 courtesy of Dr. Richard Cummings (Emory University). Prior to each experiment, lactose and mercaptoethanol were removed from this galectin-1 preparation by gel filtration on Bio-Gel P6. For solid phase binding assays of *T. vaginalis*, LPG, and CPI-GC and for galectin-1 and -3 ELISA and electrochemiluminescence assays, we used rh-galectin-1 and -3 purchased from R&D Systems. Goat polyclonal anti-galectin-1 antibody and three monoclonal antibodies against galectin-3 were obtained from R&D Systems. We confirmed by Western blot analysis using Mac2 antibody as a well established control that all monoclonal antibodies were directed against the N-terminal (non-lectin) domain of galectin-3 and did not bind the CRD domain (Fig. 2*E*).

##### Biolayer Interferometry

Biotinylated CPI-GC or LPG was prepared using Pierce EZ-Link Sulfo-NHS-LC biotinylation kit as directed by the manufacturer. Bio-layer interferometry was performed on a ForteBio Octet Red (Pall ForteBio Corp., Menlo Park, CA). Streptavidin-coated biosensors were pre-wet in PBS and loaded for 30–60 min in a solution of 50 μg/ml biotinylated CPI-GC. Following a brief wash at pH 3.0 to remove loosely associated lipid, baseline was established by incubation for 5 min in PBS followed by incubation with varying concentrations of lectins or galectins in PBS for 1 h. Dissociation was then examined by incubation for an additional hour in PBS. All steps were performed at 25 °C and at 1000 rpm (except for the loading step, which was done at 0 rpm). For galectin-3, binding was performed in PBS containing 0.005% Tween 20. Steady-state analysis was performed using the ForteBio Data Analysis software (version 6.3). Ricin and tomato lectin were purchased from Vector Laboratories (Burlingame, CA).

##### Western Blot for Galectin Binding to Whole T. vaginalis Parasites

The experimental procedure to examine the binding of galectin-1 and galectin-3 to *T. vaginalis* parasites was similar to that described for *Leishmania* (25, 26). For galectin-1 binding, *T. vaginalis* parasites (2 × 10^7^) were incubated with purified recombinant galectin-1 (∼25 μg) in 250 μl of serum-free RPMI 1640 medium containing 25 mm Hepes/PBS (1:1 ratio) in the presence and absence of lactose (100–200 mm), at 4 °C for 30 min. The concentration of NaCl in PBS was manipulated to maintain appropriate osmolarity when parasites were incubated with 100–200 mm sugar. Also, a protease inhibitor mixture was added during each incubation period to inhibit cleavage of galectin-1. Following the incubation, parasite-free supernatants were obtained by centrifugation at 4 °C at 6500 rpm for 10 min. The parasites were washed once with PBS to remove unbound galectin, then resuspended in PBS at 4 °C, followed by release of galectin-1 bound to parasites with 100 mm lactose. The supernatants of each incubation mixture were concentrated using Centricon YM-3 and analyzed by SDS-PAGE (20%), followed by Western blot analysis. Galectin-3 binding was performed in a similar manner.

##### Solid Phase Assays for Galectin Binding to Whole Parasites, LPG, and CPI-GC

Whole parasites at 3.5 × 10^5^/well fixed in 5% formalin and purified LPG or CPI-GC (0.3–5 μg, 25 μl of 80% ethanol) were added to each well of 96-well flat-bottomed microtiter plates (Corning Costar®-CARBO-BIND) and allowed to bind at room temperature for 12–18 h. The wells were washed with PBS three times and blocked with 2% BSA in PBS for 2 h. After washing the wells with PBS, galectin-1 (4 μg/well) or galectin-3 (3 μg/well) in PBS was added to each well in the absence and presence of a competing sugar (lactose) (100 mm) or a non-competing sugar (raffinose or sucrose) (100 mm) and incubated for 1.5 h at 37 °C. Wells were washed with PBS and incubated with galectin-1 and galectin-3 antibody (25 ng/well) in PBS for 2 h. After washing with PBS and three more times with PBS/Tween (0.05%), the plates were incubated with HRP-labeled donkey anti-goat IgG (Santa Cruz Biotechnology) (dilution 1:1000 in PBS-T) for 2 h. SureBlue® TMB Microwell Peroxidase Substrate (Kirkegaard & Perry Laboratories) was added to each well after a PBS/Tween wash. The reaction was stopped with 1 n HCl, and the absorbance was read at 405 nm.

##### Epithelial Cell Culture Model

Previously well characterized immortalized cell lines, originating from normal human vagina (Vk2/E6E7), endocervix (End1/E6E7), and ectocervix (Ect1/E6E7) of the human uterus (27), were cultured (28) in antibiotic-free keratinocyte serum-free medium (KSFM), supplemented with 50 μg/ml bovine pituitary extract, 0.1 ng/ml epidermal growth factor (Invitrogen), and 0.4 mm CaCl_2_ (Fisher). These cell lines have been established as a physiologically relevant *in vitro* model for the study of *T. vaginalis* pathogenesis by multiple investigators (9, 12, 17, 29–31) and have been extensively compared with their primary tissues of origin and with primary organotypic cultures showing no significant differences in responses to *T. vaginalis* parasites and purified LPG as well as other innate immunity ligands (9, 11, 13, 27, 28, 32–36). VEC-100^TM^ tissues (MatTek, Ashland, MA) were handled as described. For epithelial *Trichomonas* infection experiments, *Trichomonas* was added at a multiplicity of infection of 1:10 to epithelial monolayers. After 6–24-h incubations under anaerobic conditions mimicking the vaginal microenvironment (Mitsubishi AnaeroPack, Fisher), supernatants were collected for assessment of immune mediator levels, and cells were used for viability assessment as described. LPG and CPI-GC were used at a dose of 240 μg/ml, which is equivalent to typical parasite burden and is both non-toxic and immunostimulatory as determined before in dose-range studies (12).

##### Immunofluorescent Staining and Western Blot for Galectin Expression by Human Cervical and Vaginal Epithelial Cells

Epithelial cells, grown on glass chamber slides (34), were fixed in absolute ethanol for 20 min, blocked in PBS, 1% BSA, incubated with 5 μg/ml goat anti-galectin-1, anti-galectin-3 IgG, or IgG control (R&D Systems) for 20 min, washed three times in PBS, and exposed to 12.5 μg/ml donkey anti-goat Texas Red-conjugated antiserum (Jackson ImmunoResearch, West Grove, PA) for 20 min. Results were read by Olympus BX60 microscope (Olympus America, Melville, NY) and Image-Pro Plus (Media Cybernetics, Silver Spring, MD). For Western blot, endocervical, ectocervical, and vaginal epithelial cells were grown to confluence in 6-well plates and lysed in Tris lysis buffer supplemented with protease inhibitors and followed by total protein determination by the Pierce BCA protein assay (Fisher). The cell lysates (15 μg of total protein for the galectin-3 Western blot and 25 μg of total protein for the galectin-1 Western blot) were fractionated by SDS-PAGE and transferred to a PVDF membrane. The membranes were washed and blocked with Tris buffer with 0.1% Tween 20 and 5% nonfat dry milk. The membranes were then incubated with anti-galectin-1 rabbit polyclonal antibody (SC-28148), and after a washing step with an anti-rabbit HRP-conjugated antibody (SC-2020) or with anti-galectin-3 goat polyclonal antibody (SC-19283) followed by a donkey anti-goat HRP-conjugated antibody (SC-2004). All reagents were from Santa Cruz Biotechnology, Dallas, TX. After the unbound secondary antibody was washed out, the membranes were exposed to substrate and the bands visualized by x-rays.

##### Generation of siRNA Galectin Knockdown Clones

Small interfering RNA (siRNA) was used for gene-specific RNA interference to block the expression of human galectin-1 and galectin-3. Short hairpin RNAs (shRNAs) that generate siRNA intracellularly were expressed using MISSION shRNA clones (GenBank^TM^ accession numbers NM_002305.2-368s1c1, NM_002305.2-149s1c1, NM_002305.2-95s1c1, NM_002305.2-308s1c1, NM_002305.2-239s1c1, NM_002306.1-664s1c1, NM_002306.1-498s1c1, NM_002306.1-442s1c1, NM_002306.1-675s1c1, and NM_002306.1-536s1c1) within the lentivirus vector pLKO.1-Puro, which contains both bacterial (ampicillin) and mammalian (puromycin) resistance genes (Sigma). The shRNA plasmid vectors were prepared from bacterial glycerol stocks (Sigma) after bacterial culture amplification in LB Broth (Sigma) containing 100 μg/ml ampicillin and Endofree plasmid purification maxi kit per the manufacturer's instructions (Qiagen, Valencia, CA). Cell transfection was performed as published (9). Stable gene silencing was acquired by selection of transfected immortalized epithelial cells (End1/E6E7) with 10 μg/ml puromycin (Sigma). The non-target MISSION shRNA control vector (Sigma) was used for generation of a control End1/E6E7 cell line. The control vector shRNA sequence contains 5-bp mismatches to any known human and mouse gene and activates RISC and the RNAi pathway but does not target any human or mouse gene. Gene expression silencing was confirmed by Western blot and ELISA (R&D Systems).

##### Quantification of Immune Mediators

Protein levels of galectin-1, galectin-3, IL-8, RANTES, and MIP-3α were simultaneously quantified using a custom-designed multiplex electrochemiluminescence (ECL) immunoassay, Sector Imager 2400 and Discovery Workbench Software (Meso Scale Discovery, Gaithersburg, MD), validated against traditional ELISA (9, 37, 38). Galectin-1 and -3 levels were also assessed by traditional commercially available ELISA (R&D Systems). The concentrations of soluble extracellular mediators released in the cell culture supernatants were measured in picograms/ml. The galectin levels in cell lysates (cell-bound galectins) were measured also in picograms/ml by ELISA and then normalized to the total protein of cell lysate, thus quantifying galectins in nanograms/mg total protein. Total protein levels were measured using the Pierce BCA protein assay. The ELISA and the BCA assays were read using a Victor2 reader (PerkinElmer Life Sciences).

##### Cell Viability Assays

Epithelial cell viability in the presence of LPG, CPI-GC, and recombinant galectins was ascertained by the non-radioactive CellTiter96 MTT assay, which converts the MTT dye into a blue formazan product (Fisher). Cell viability of *T. vaginalis*-infected cells was assessed microscopically and by the trypan blue exclusion assay (Fisher) because *Trichomonas* can convert the MTT dye and interferes with the MTT assay.

##### Statistics

Data were analyzed by ANOVA (GraphPad Prism, version 5.0; GraphPad Software). *p* values <0.05 were considered significant.

## Results

### 

#### 

##### Galectin-1 and -3 Are Expressed and Secreted by Human Vaginal and Cervical Epithelial Cells

*T. vaginalis* is uniquely adapted to the human host and the vaginal mucosal environment where it survives for a long time as an extracellular parasite adherent to the epithelial cells. However, no studies have addressed the release and regulation of galectin-1 and -3 by the epithelial cells representing the three anatomic compartments (vagina, ectocervix, and endocervix of the uterus) contributing to the lower female genital tract barrier. Because of the strict species-specificity of this protozoan parasite, *in vitro* modeling is best suited for the study of the causality of molecular signaling in *T. vaginalis* infection.

**FIGURE 1.**
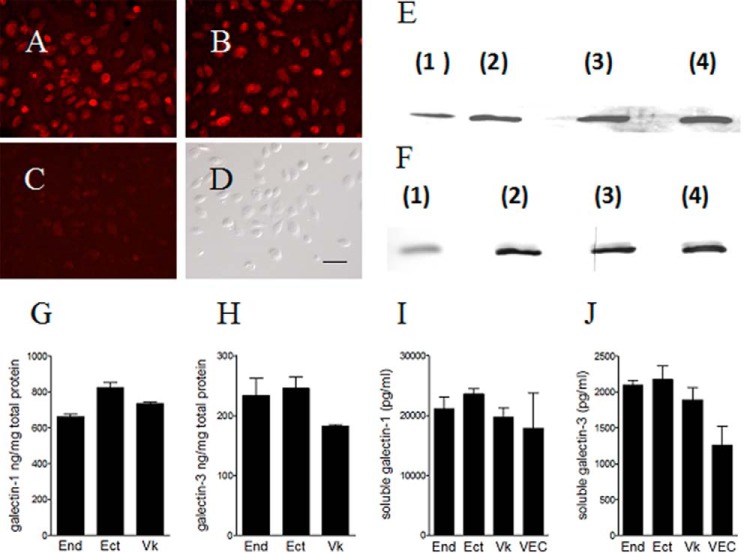
**Expression of galectin-1 and -3 by human cervicovaginal epithelial cells.** *A–D,* Texas Red immunofluorescent visualization of galectin-1 (*A*) and galectin-3 (*B*) in ectocervical (*Ect*) cells with isotype IgG control in *C* and matched bright field in *D. E* and *F,* Western blot analysis of galectin-1 (*E*) and galectin-3 (*F*) in epithelial cell lysates loaded at equal total protein/lane (25 μg for galectin-1 and 15 μg for galectin-3) as follows: *lane 1* = recombinant galectin control; *lane 2* = endocervical (*End*); *lane 3* = ectocervical; and *lane 4* = vaginal (*Vk*) cells. *G–J,* cell-associated (*G* and *H*) and soluble (*I* and *J*) protein levels of galectin-1 and -3 expressed by endocervical, ectocervical, vaginal cells and *in vitro* reconstructed three-dimensional ectocervical tissues (*VEC*), measured by immunoenzyme assays. *Bars* are mean and S.E. Picograms of galectin/ml of cell culture supernatant or nanograms of galectin/mg of total protein in cell lysates from at least three experiments with each cell line are shown.

To address the gaps in current basic knowledge of *Trichomonas* pathobiology, we first determined whether human vaginal, ectocervical, and endocervical epithelial cells express and release galectin-1 and -3. We used a well establish immortalized epithelial cell line model closely resembling the normal phenotypes of the human vaginal and cervical tissues of origin and the pathophysiologic responses to infection of paternal primary cells (9, 11, 27, 32, 33). Both galectins were detected by immunofluorescence (Fig. 1, *A* and *B*) and Western blot (Fig. 1, *E* and *F*) and quantified by ELISA in cell lysates (Fig. 1,*G* and *H*) and cell supernatants (Fig. 1, *I* and *J*) from the vaginal, ectocervical, and endocervical epithelial cells. Similar secreted galectin levels were measured in supernatants from all three immortalized cell lines compared with vaginal-ectocervical tissues reconstructed from primary cells (Fig. 1, *I* and *J*), thus warranting the further use of the epithelial cell lines as a highly reproducible model for mechanistic analyses of causality under isogenic conditions.

##### Wild Type T. vaginalis Protozoa, LPG, and CPI-GC Bind to Human Galectin-1 and Galectin-3

**FIGURE 2.**
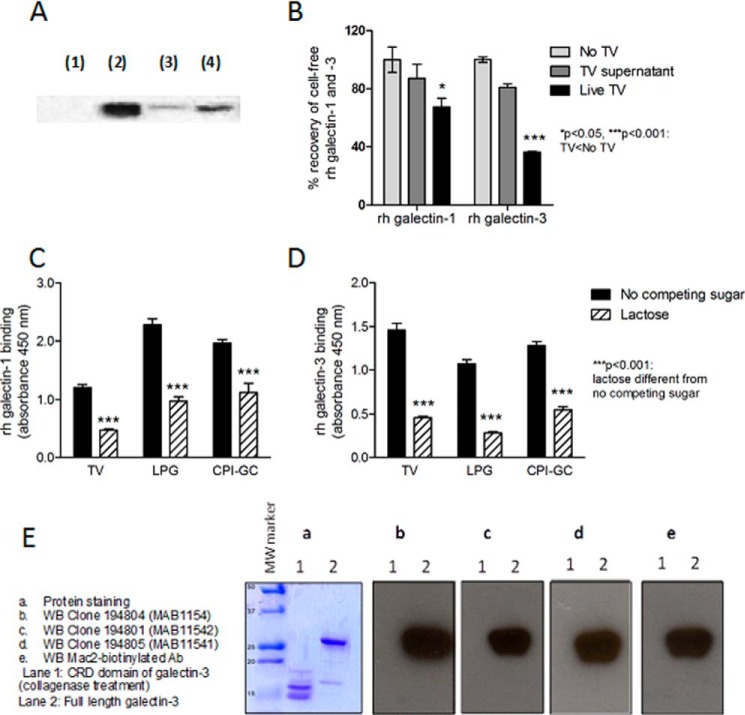
**rh-galectin-1 and -3 binding to *T. vaginalis* (*TV*) and purified *T. vaginalis* LPG and CPI-GC.** *A* and *B,* live *T. vaginalis* bound to galectin-1 and -3 in solution, demonstrated by Western blot (*A*) and ELISA (*B*). *A, lane 1, T. vaginalis* alone; *lane 2, T. vaginalis* bound to galectin-1; *lane 3, T. vaginalis* bound to galectin-1 after elution with 100 mm lactose, and *lane 4,* galectin-1 alone (75 ng). *C* and *D,* galectin-1 and -3 bound to *T. vaginalis*, LPG and CPI-GC fixed on solid phase, detected by ELISA in the presence of 100 mm lactose or no competing sugar. *Bars* are means and S.E.; *p* values are from two-way ANOVA and Bonferroni multiple comparison post-test. *E,* Western blot (*WB*) analysis demonstrating that all galectin-3 monoclonal antibodies used in the experiments shown in *A–E* bind the N-terminal (non-lectin) domain of galectin-3.

We next examined whether soluble (rh) galectin-1 and galectin-3 bind to live *T. vaginalis* (Fig. 2, *A* and *B*) or to fixed whole protozoa, purified LPG, and CPI-GC, attached to a solid phase (Fig. 2, *C* and *D*).

Results from Western blot analysis showed that galectin-1 bound to live *T. vaginalis* (clinical isolate UR1), and this binding, visualized by Western blot, was significantly reduced by subsequent dissociation of galectin-1 by 100 mm lactose, suggesting a role of β-galactoside in the *Trichomonas*-galectin-1 interaction (Fig. 2*A*). Galectin-3 associated with live *Trichomonas* was also detected by Western blot; however, the Western blot band was not significantly reduced after subsequent exposure to lactose even though some amount of free galectin-3 was released and detectable by ELISA (data not shown). These differences in the competitive release of galectin-1 and -3 upon lactose treatment detectable by Western blot might be due to the preferential binding of galectin-3 to internal LacNAc motifs within the extended polyLacNAc repeats and the low potency of lactose as a competitive antagonist for galectin-3 (*K_d_* for lactose is 26 μm whereas *K_d_* for LacNAc3 is 0.35 μm). Upon binding to glycans, the galectin-3 molecules form pentamers, which exhibit significantly higher avidity and affinity than galectin-1, which is a dimer with more than 50-fold lower affinity for polylactosamine than galectin-3 (39–41).

The binding of live *Trichomonas* to galectin-1 and -3 in solution was further confirmed by estimating levels of galectins that remained unabsorbed by the parasite (Fig. 2*B*). Live parasites were incubated with galectins for 24 h, and their concentrations were measured by a well standardized ELISA (R&D Systems) after depleting the parasites for 15 min at 1000 × *g*. The recovery of galectins from the solution was compared with that following incubation in medium alone or in conditioned medium that was prepared after 24 h of incubation with *Trichomonas*. The results (shown for the laboratory wild type strain B7RC2) were similar among several primary clinical isolates tested, confirming that direct contact with the parasite contributed to the reduction of galectin levels in the medium.

**FIGURE 3.**
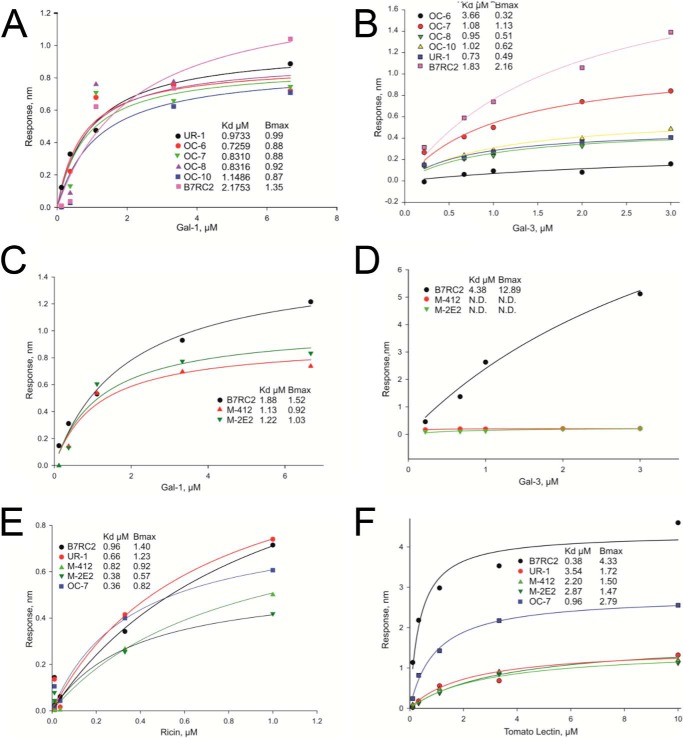
**Kinetics of CPI-GC galectin binding.** *A–F,* kinetic curves of CPI-GC binding to recombinant human galectin-1 (*A* and *C*), galectin-3 (*B* and *D*), and the plant lectins ricin (*E*) and tomato lectin (*F*) evaluated by bio-interferometry. CPI-GCs were prepared from different primary clinical *T. vaginalis* isolates (UR-1, OC-6, OC-7, OC-8, and OC10), the wild type laboratory strain B7RC2 and its mutant derivatives M-412 and M-2E2.

Galectin binding to fixed *Trichomonas*, LPG, and CPI-GC was tested by ELISA where galectins and lactose or no-competing sugar (raffinose or sucrose) were added simultaneously and allowed to compete for the parasite's carbohydrate motifs (Fig. 2, *C* and *D*). In contrast to the Western blot experiments with live parasites, where lactose was added sequentially after the galectin complex with live *Trichomonas* was already formed, in the ELISA setting lactose inhibited the binding of both galectin-1 (Fig. 2*C*) and -3 (Fig. 2*D*) to fixed parasites thus confirming a β-galactoside participation in the recognition by both galectins. The findings were consistent with the presence of β-galactoside, lactosamine (Galβ→GlcNAc) in CPI-GC, which was previously suggested by endo-β-galactosidase treatment and mass spectrometry (12). The B7RC2 *T. vaginalis* strain also showed lactose-dependent binding to recombinant galectin-1 and -3 assessed by ELISA (data not shown). The competitive inhibition of galectin-3 binding by lactose in the ELISA format can be explained by the fact that in these experiments the protozoan cells were fixed in formalin before the galectin was added, and thus lipid raft clustering of the LPG molecules could not occur due to the alcohol present in formalin, creating perhaps a weaker interaction more susceptible to lactose displacement as compared with the interactions observed with live protozoa in the Western blot experiments.

##### CPI-GCs from Various T. vaginalis Isolates and Mutants Differ in Binding Affinity to Galectin-1 and Galectin-3

To further understand the kinetics of galectin-1 and -3 binding to the functional CPI-GC domain of the LPG molecule, we used bio-layer interferometry comparing CPI-GCs from various *T. vaginalis* clinical isolates (UR1 and OC-6–8 and -10) as well as CPI-GC from the B7RC2 laboratory strain and B7RC2-derived mutants (M-412 and M-2E2) with truncated LPGs. In this assay, the dissociation constant (*K_d_*) is an inverse measure of galectin-ligand avidity, and *B*_max_ is a measure of the maximum concentration required for receptor-ligand equilibrium. The functional affinity can be expressed as *B*_max_/*K_d_* values. Interestingly, although all tested CPI-GCs from clinical isolates and the wild type of the laboratory strain B7RC2 bound to galectin-1 with a relatively narrow range of binding affinities (*B*_max_/*K_d_* varied from 0.62 to 1.21) (Fig. 3*A*), a diversity with over a log range was observed in galectin-3 binding (*B*_max_/*K_d_* from 0.087 to 1.18) (Fig. 3*B*). In comparison with the WTB7RC2, the mutant (m) CPI GCs M-4.12 and M-2E2 showed preserved albeit lower affinity to galectin-1 (Fig. 3*C*) but no detectable binding to galectin-3 (Fig. 3*D*).

CPI-GC monosaccharide analysis showed substantial divergence between the clinical isolates and expected differences between the WTB7RC2 and the two mutants (Table 1). Similarly to compositions published for LPGs from the WTB7RC2 and the two mutant derivatives (30), the mutant CPI-GCs showed reduced Gal and GlcN in comparison with the WT CPI-GC. Even though the reduced Gal and GlcN content was consistent with the reduced mutant binding to galectin-1 and -3, the differences in monosaccharide composition, including variations in Gal and GlcN content, observed between the WT CPI-GCs from individual clinical isolates did not directly correlate and could not explain the spread and diversity in galectin-3 affinity. Therefore, we postulated that availability of non-reducing terminal β-galactoside and poly-LacNAc would be a better predictor of binding affinity than simply monosaccharide content. To test that premise, we compared the CPI-GCs by their affinity to ricin and tomato lectin (*Lycopersicon esculentum* agglutinin (LEA)). Ricin specifically recognizes glycans containing a non-reducing terminal β-gal attached to GlcNAc, whereas LEA has high affinity for linear polyLacNAc, for which galectin-3 shows high affinity (42, 43). The kinetics of ricin binding mimicked the patterns observed with galectin-1 showing similar binding of wild type CPI-GCs and measurable but reduced binding of the CPI-GC mutants (Fig. 3*E*), confirming the presence of non-reducing terminal β-galactoside in all CPI-GCs. The binding to LEA mimicked the patterns observed with galectin-3, showing a similar spread of affinities for the wild type CPI-GCs and significantly reduced binding of the mutant CPI-GCs (Fig. 3*F*). Thus, the differences in LEA binding supported the notion that the reduced number of polyLacNAc and/or modification that masks the LacNAc-binding sites may be contributing to a structural diversity related to the interaction with galectin-3 (but not galectin-1) among the clinical isolates and mutant derivatives.

**TABLE 1:** **Monosaccharide composition of CPI-GC purified from *T. vaginalis* clinical isolates (UR-1, OC-6, OC-7, OC-8, OC-9, and OC-10), a wild type laboratory strain (B7RC2), and its mutant derivatives (M-2E2 and M-412)**

CPI-GC	Rha		GalN		Gal		GlcN		Glc		Xyl	
	*nmol*	%	*nmol*	%	*nmol*	%	*nmol*	%	*nmol*	%	*nmol*	%
UR-1	50.312	21.1	4.934	2.37	68.518	39.08	57.032	30.81	4.313	2.91	20.451	3.72
OC-6	4.901	26.11	5.472	29.15	3.656	19.48	2.954	15.74	0.803	4.28	0.985	5.25
OC-7	9.500	24.80	1.316	3.44	14.573	38.05	8.160	21.30	1.374	3.59	3.375	8.81
OC-8	18.393	33.90	10.286	18.96	13.906	25.63	5.888	10.85	3.511	6.47	2.280	4.20
OC-10	30.618	42.05	ND*^a^*	ND*^a^*	22.655	31.12	16.547	22.73	2.552	3.50	0.435	0.60
B7RC2	40.548	20.69	4.500	2.30	52.757	26.92	69.335	35.38	8.658	4.42	20.179	10.30
M-2E2	65.224	42.10	5.604	3.62	9.359	6.04	45.732	29.52	13.121	8.47	15.888	10.26
M-412	43.734	25.51	39.093	22.80	15.508	9.04	33.433	19.50	18.281	10.66	21.407	12.48

*^a^* ND means not detectable.

Our unpublished mass spectrometry (MS) data (not shown in detail here) obtained in Catherine Costello's Core Facility at Boston University provide additional evidence for the importance of lactosamine repeats in the kinetics of galectin-1 and -3 binding. MALDI-TOF detected a peak at *m*/*z* 8160.7 in the CPI-GC purified from the wild type BC7RC2, whereas corresponding peaks of the mutant CPI-GCs M-4.12 and M-2E2 were observed at *m*/*z* 6384.2 and *m*/*z* 6381.1, respectively, with evidence for lactosamine repeats in the wild type CPI-GC only.

##### T. vaginalis Regulates Soluble (Extracellular) and Cell-associated Galectin-1 and -3 Levels in the Human Cervicovaginal Epithelium

We next examined whether binding to *Trichomonas* changes soluble/extracellular levels of galectin-1 and -3 as expected from the *ex vivo* binding experiments with recombinant proteins and whether reducing the levels of one galectin affects the expression levels of the other.

**FIGURE 4.**
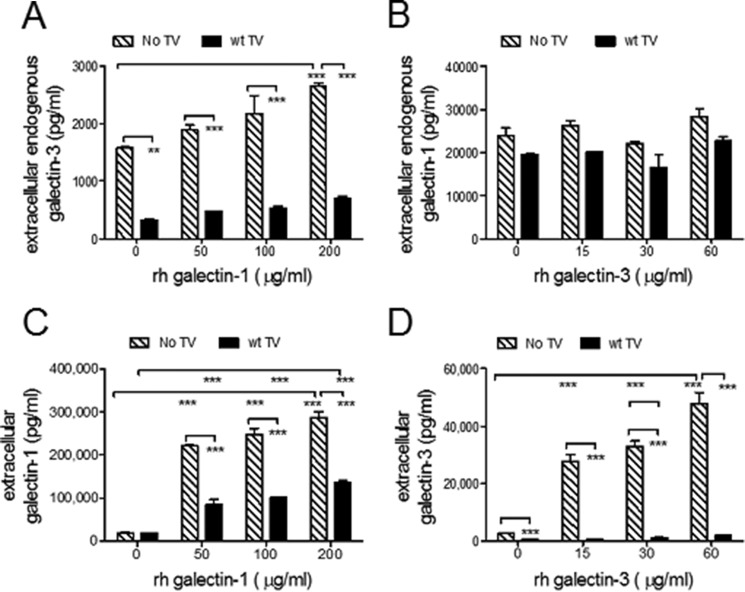
**Regulation of soluble galectin-1 and -3 in the human cervical epithelial space by trichomonas.** Cervical epithelial cells were infected with *Trichomonas* (wild type strain B7RC2) in the presence of escalating doses of rh-galectin-1 (*A* and *C*) or galectin-3 (*B* and *D*). The *x* axis on each plot shows concentration of rh-galectin-1 or galectin-3 added to the infection model. The *y* axis shows extracellular levels of galectins quantified in the cell culture supernatants by ELISA after 24 h of incubation with *Trichomonas* and the particular recombinant galectin. *A* and *B* show the levels of endogenous galectin-3 and -1 found in the medium after the treatment with exogenously added galectin-1 and -3, respectively. *A,* only endogenous galectin 3 was available for measure because no exogenous rh-galectin-3 was added. The endogenous galectin-3 in this case went up as a result of stimulating the cultures with escalating doses of recombinant galectin-1. Similarly, in *B* we show endogenous galectin-1 measured when no exogenous galectin-1 was added. In contrast, *C* and *D* show both the endogenous and the exogenous galectin-1 or -3 levels, respectively. *p* values are from ANOVA and Bonferroni multiple comparison test. **, *p* < 0.01; ***, *p* <0.001; two-way ANOVA, Bonferroni multiple comparisons test. *Bars* represent mean and S.E. from duplicate cultures in one of three experiments.

First, we stimulated cervical epithelial cells with escalating doses of recombinant galectin-1 or -3 in the presence or absence of *Trichomonas* (B7RC2) infection and measured galectin levels in 24-h cell culture supernatants. In the absence of infection, recombinant galectin-1 increased the extracellular levels of endogenous galectin-3, *p* < 0.001 (Fig. 4*A*), while recombinant galectin-3 had no effect on endogenous galectin-1 levels (Fig. 4*B*). *Trichomonas* significantly reduced the endogenous levels of galectin-3 in both the absence (*p* < 0.01) or presence of recombinant galectin-1 (*p* < 0.001) (Fig. 4*A*) but did not significantly decrease the levels of endogenous galectin-1, which were expressed by the cervical epithelial cells of at least 10-fold higher levels than galectin-3 (Fig. 4*B*). Furthermore, *Trichomonas* reduced the levels of the recombinant galectin-1, *p* < 0.001 (Fig. 4*C*), and almost entirely depleted galectin-3 from the cell culture supernatants, even at the highest dose of the recombinant protein supplement, *p* < 0.001 (Fig. 4*D*).

**FIGURE 5.**
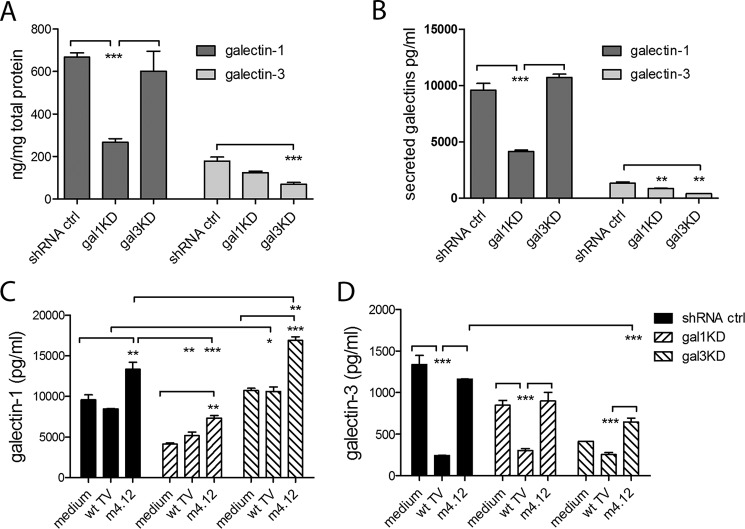
**Effect of siRNA knockdown on extracellular and cell-associated levels of galectin-1 and -3 and effect of *Trichomonas* on cell-free levels of both galectins.** *A,* cell-associated levels per total cellular protein content in endocervical epithelial clones stably transfected with shRNA control (*ctrl*) or siRNA for galectin-1 knockdown (*gal-1KD*) or galectin-3 knockdown (*gal-3KD*); *B,* secreted extracellular levels of galectin-1 and -3 in the shRNA control, gal-1KD, and gal-3KD supernatants; *C* and *D*, cell-free levels of secreted galectins following infection for 24 h with *T. vaginalis* wild type (*WT TV*) laboratory strain B7RC2 and its mutant derivative m4.12. *p* values are from ANOVA and Bonferroni multiple comparisons test. *Bars* represent mean and S.E. from five experiments in *A* and three experiments in *B–D*. *, *p* < 0.05; **, *p* < 0.01; ***, *p* <0.001; two-way ANOVA, Bonferroni multiple comparisons test.

Next, to further confirm the ability of galectin-1 to regulate galectin-3 expression, we knocked down endogenous galectin-1 levels in the endocervical epithelial cells using small interfering short hairpin (sh)RNA, which was effective in significantly decreasing both secreted and cell-associated protein levels (Fig. 5, *A* and *B*). Indeed, knocking down galectin-1 also reduced levels of extracellular endogenous galectin-3 (Fig. 5*B*), which supported the results obtained with recombinant galectin-1. The wild type B7RC2 did not significantly affect the endogenously high galectin-1 levels in the shRNA control and gal-3KD clones (Fig. 5*C*), although it significantly depleted the secreted levels of galectin-3 in both the shRNA control and galectin-1KD clones (Fig. 5*D*). The mutant *Trichomonas* (m4.12), which showed reduced affinity for galectin-1 (Fig. 3*C*) and complete loss of affinity for galectin-3 (Fig. 3*D*), did not affect levels of galectin-3 (Fig. 5*D*), although it increased the extracellular levels of galectin-1 (Fig. 5*C*). These findings suggest that the depletion of galectin-3 by the parasite is dependent on binding affinity. The increase of galectin-1 in the cell culture supernatant could be explained by the fact that in comparison with the wild type B7RC2, the mutants show reduced adherence to the epithelial cells (17), although as we show they still bind galectin-1, albeit with lower affinity (Fig. 3*C*), thereby keeping more of galectin-1 in the extracellular space. It remains to be further investigated whether *Trichomonas* can stimulate the expression of galectin-1, which would be masked by the absorption of the extracellular levels by the wild type parasite in this experimental setting.

**FIGURE 6.**
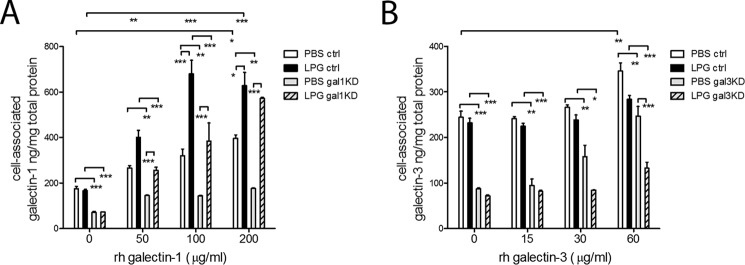
**LPG regulation of cell-associated galectin-1 and -3 in the human cervical epithelial space.** Control (*shRNA ctrl*) and galectin knockdown epithelial cell clones were treated for 24 h with rh-galectin-1 (*A*) or galectin-3 (*B*) in the presence of *T. vaginalis* (*wild type*) lipophosphoglycan (*LPG*) or vehicle control (medium or PBS), and the levels of cell-associated galectins (both endogeneous and exogenous) were determined. *p* values are from ANOVA and Bonferroni multiple comparisons test. *Bars* represent means and S.E. from duplicate cultures in one of three experiments. *, *p* < 0.05; **, *p* < 0.01; ***, *p* <0.001; two-way ANOVA, Bonferroni multiple comparisons test.

We have previously shown that *T. vaginalis* adherence to the cervicovaginal epithelial cells is LPG-dependent (9). Therefore we examined the role of LPG in delivering the soluble galectins to the epithelial cells. Purified wild type LPG was added to the epithelial cells along with escalating doses of recombinant galectin-1 or galectin-3 and cell-associated galectin levels were measured in cell lysates after removing the extracellular medium (Fig. 6). Adding escalating doses of recombinant (exogenous) galectin-1 or -3 to the epithelial cells in the absence of LPG weakly increased the concentrations of both galectins in the cell lysates with the highest dose only (*p* < 0.05, for galectin-1, Fig. 6*A*, and *p* < 0.01 for galectin-3, Fig. 6*B*) suggesting limited abilities of the epithelial cells to capture the soluble galectins on their own. The recombinant galectin-3 but not the recombinant galectin-1 restored the wild type phenotype of the galectin knockdown epithelial cells, suggesting a more efficient uptake of galectin-3 than galectin-1.

LPG had profound and opposing effects on the capture of galectin-1 and -3 by the epithelial cells. When added together with recombinant galectin-1, LPG increased the levels of cell-associated galectin-1 by over 2-fold, *p* < 0.001 (Fig. 6*A*) The increase of galectin-1 levels in the cell lysates in the presence of LPG was observed also in gal-1KD cells, which confirmed that the increase was due to capturing the exogenous galectin rather than stimulation of new galectin-1 protein synthesis. A possible interpretation of these findings is that LPG bound simultaneously to the epithelial cell surface and the recombinant galectin-1 and in this way mediated the galectin-1 association with the epithelial cells. Thus, we are underestimating the bioavailability of galectin-1 in the presence of whole parasites when using supernatants and not measuring its levels absorbed by the parasites that are firmly attached to the epithelial surface. Indeed, in the absence of LPG, recombinant galectin-1 did not restore cell-associated levels in the gal-1KD cells.

The opposite phenomenon was observed when recombinant galectin-3 was added in the presence of LPG (Fig. 6*B*). The presence of LPG actually prevented the capture of the highest dose of recombinant protein by the shRNA control epithelial cells, *p* < 0.01, and the gal-3KD cells, *p* < 0.001. In fact, the presence of LPG prevented the recombinant proteins from restoring the cell-associated levels of galectin-3 in the gal3-KD cells. It is possible that the interaction of LPG with oligomerized galectin-3 prevented epithelial cell uptake by keeping the galectin in the extracellular space. It is also possible that LPG binding to the epithelial cells mediated degradation of galectin-3 by host enzymes, thus resulting in decreased cell-associated levels of this galectin. This potential mechanism warrants further studies and is supported by our findings that mutant *Trichomonas* that lacked binding to galectin-3 and adherence to epithelial cells showed diminished abilities to decrease extracellular levels of galectin-3 (Fig. 5*D*).

##### T. vaginalis Evades Host Immunity via Galectin-1 and -3 Binding

We had previously shown that *T. vaginalis* LPG and CPI-GC trigger proinflammatory responses by the cervicovaginal epithelial cells, including the chemokines IL-8 and MIP-3α (9, 12). Our findings of LPG/CPI-GC binding to galectin-1 and -3 suggested that these two galectins may mediate the CPI-GC effects on human host immunity.

**FIGURE 7.**
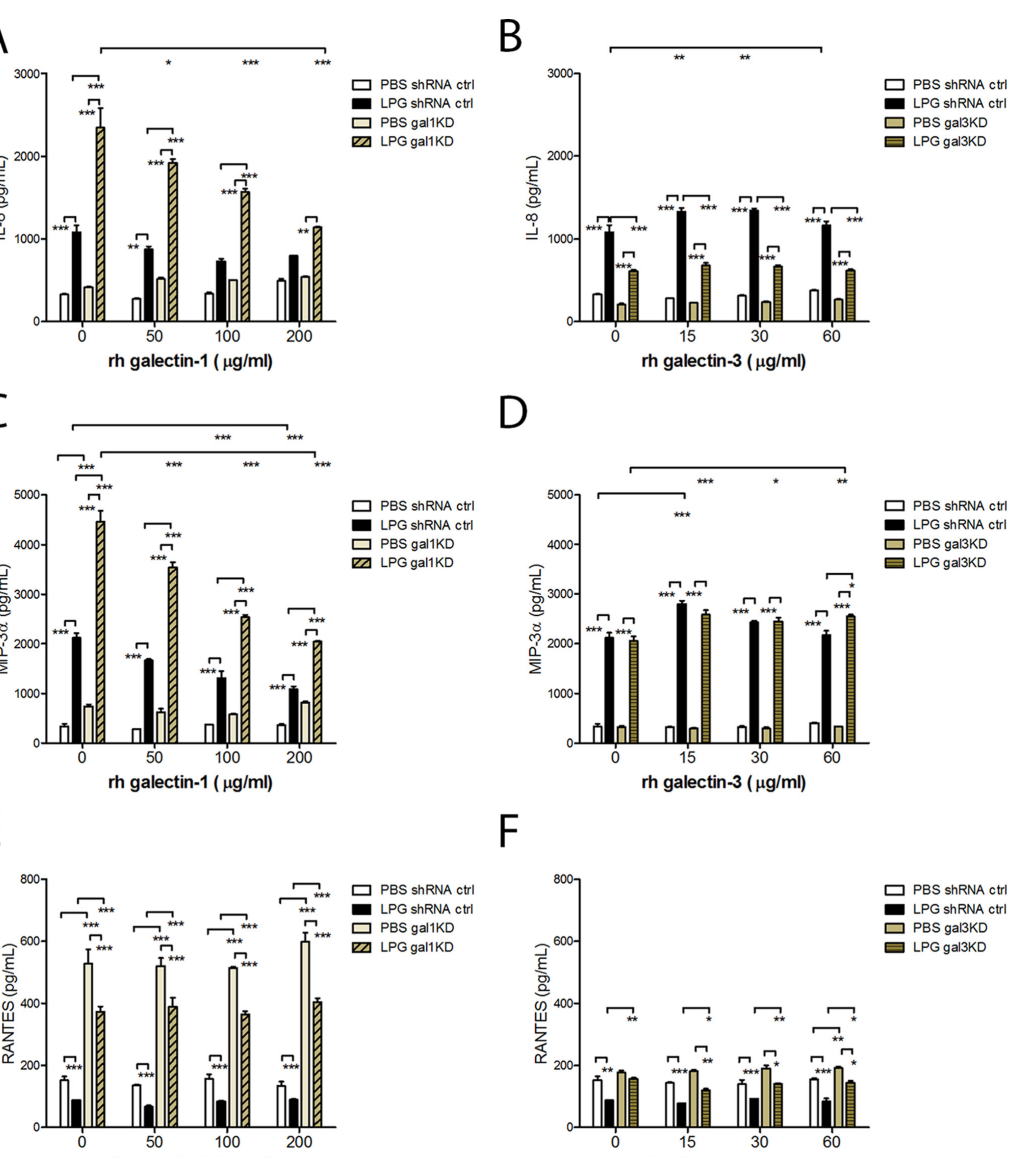
**Immune regulation by endogenous and exogenous galectin-1 and galectin-3.** Control (*shRNA ctrl*) and galectin knockdown endocervical epithelial cell clones were treated for 24 h with escalating doses of rh-galectin-1 (*A, C,* and *E*) or galectin-3 (*B, D,* and *F*) in the presence of *T. vaginalis* (wild strain B7RC2) lipophosphoglycan (*LPG*) or vehicle control (medium or PBS). The chemokine production was assessed in cell culture supernatants. *Bars* are means and S.E. from duplicate cultures representing three experiments. *, *p* < 0.05; **, *p* < 0.01; ***, *p* <0.001; two-way ANOVA, Bonferroni multiple comparisons test.

We first compared the effect of LPG on the expression of proinflammatory chemokines by the control and galectin knockdown epithelial clones (Fig. 7). The knockdown of galectin-1 increased, although the knockdown of galectin-3 decreased the production of IL-8 in response to LPG (Fig. 7, *A* and *B*). The MIP-3α production in response to LPG was also significantly increased in the galectin-1 knockdown cells (Fig. 7*C*) but was not affected by the knockdown of galectin-3 (Fig. 7*D*) suggesting alternative pathways for IL-8 and MIP-3α stimulation. Our results suggested that the affinity of *T. vaginalis* LPG to galectin-1 may be essential for the parasitic survival in the human host.

We next examined whether adding exogenous galectins may restore the normal immune response to LPG in the galectin knockdown cells. Adding escalating doses of recombinant galectin-1 to the gal-1KD cells restored the original phenotype of LPG-induced suppression of chemokine expression in a dose-dependent fashion (Fig. 7, *A* and *C*). The more galectin-1 was added, the lower the IL-8 or MIP-3α responses to LPG were. Adding exogenous galectin-3 did not improve the suppressed IL-8 response in the galectin-3 knockdown cells (Fig. 7*B*), which was consistent with the previously observed interference of LPG with galectin-3 uptake by the epithelial cells (Fig. 6*B*) and suggested dependence of the IL-8 production on cell-associated or intracellular rather than soluble/extracellular galectin-3, which needs to be further investigated.

In contrast to its stimulating effects on IL-8 and MIP-3α, LPG suppressed the epithelial cell output of RANTES (Fig. 7, *E* and *F*). RANTES was up-regulated by galectin-1 knockdown (Fig. 7*E*) and not affected by galectin-3 knockdown (Fig. 7*F*), supporting the opposing roles of these two galectins in the cervicovaginal epithelial cells. RANTES was suppressed by LPG under all conditions (Fig. 7, *E* and *F*). Collectively, the experiments with galectin-1 and galectin-3 knockdown clones supported our hypothesis that galectin-1 and -3 play opposing roles in controlling the immune responses to *T. vaginalis* infection and that *T. vaginalis* may exploit LPG binding to both galectins as a mechanism to alter host immunity.

**FIGURE 8.**
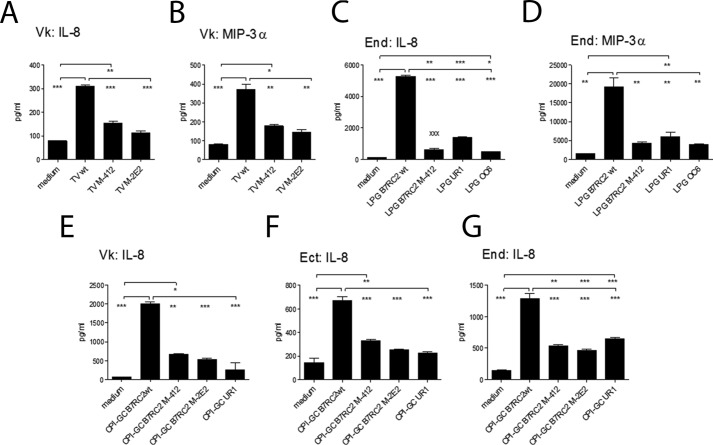
**Chemokine responses to *T. vaginalis* (*TV*) parasites, LPG, and CPI-GC from laboratory strains and clinical isolates that showed different affinity to galectin-3.** IL-8 and MIP-3α were quantified by Meso Scale Discovery multiplex in 24-h supernatants from endocervical (*End*), ectocervical (*Ect*), or vaginal (*Vk*) epithelial cells after stimulation with live parasites (*A* and *B*), purified LPG (*C* and *D*), or CPI-GC (*E–G*) from wild type (*wt*) B7RC2, mutants (M-4.12 and M-2E2) and clinical isolates UR1 and OC6. *Bars* represent means and S.E. from duplicate or triplicate cultures in three experiments performed with each cell line. *, *p* < 0.05; **, *p* < 0.01; ***, *p* <0.001; two-way ANOVA, Bonferroni multiple comparisons test.

We next hypothesized that the immune responses elicited by *T. vaginalis* depend on the polyLacNAc-driven affinity/avidity of LPG/CPI-GC binding to galectin-1 and -3. To address this question, we compared immune responses to LPG and CPI-GC purified from wild type and mutant B7RC2 *Trichomonas* (Fig. 8, *A* and *B*) as well as LPG and CPI-GC (Fig. 8, *C* and *D*) purified from the clinical isolates that showed lowest affinity to galectin-3 by biointerferometry, *e.g.* UR1 and OC6 (Fig. 8). Both LPG (Fig. 8, *A* and *C*) and CPI-GC (Fig. 8, *E–G*) from the B7RC2WT strain, which showed strong affinity to both galectin-1 and -3 (Fig. 3, *A* and *B*), induced higher levels of IL-8 than LPG or CPI-GC from the mutants (M4–12 and M2E2), which had weaker but relatively preserved binding to galectin-1 and lacked binding affinity to galectin-3 (Fig. 3, *C* and *D*). The same was true for MIP-3α (Fig. 8, *B* and *D*). Similarly, LPG or CPI-GC from OC6 and UR1 induced lower levels of chemokines compared with the B7RC2WT strain (Fig. 8, *C–G*), supporting the proposed dependence of these responses on the relative affinity to galectin-3. These differences in the proinflammatory activities of the wild type and mutant CPI-GC were observed in epithelial cells from all three anatomic compartments of the female genital tract, endocervical, ectocervical, and vaginal (Fig. 8, *E–G*).

**FIGURE 9.**
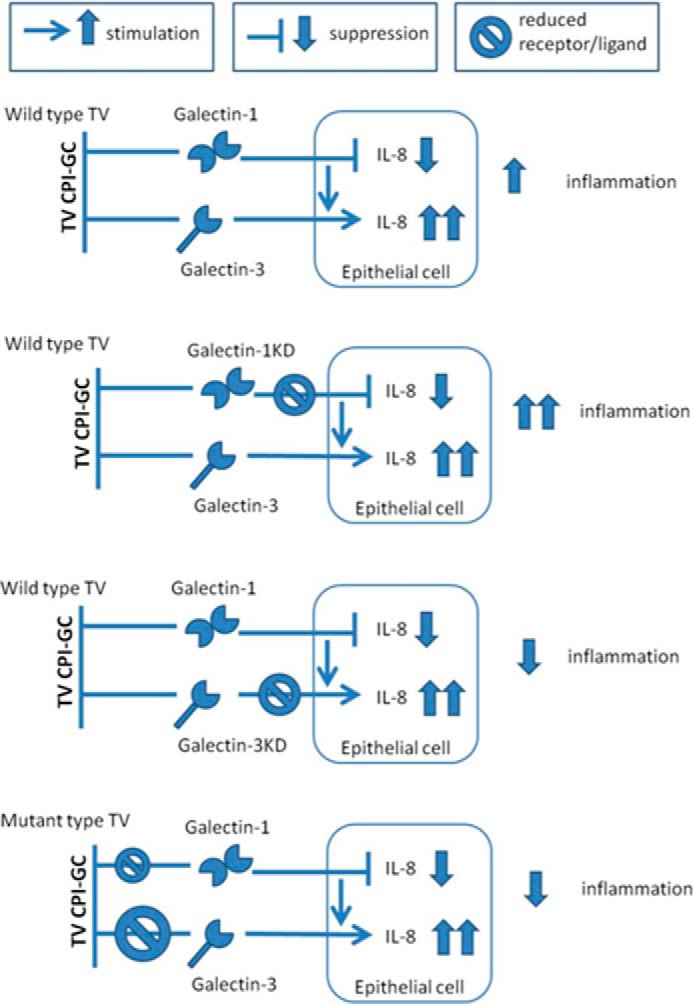
**Schematic summary of the different scenarios of *T. vaginalis* signaling to the human epithelial cells via LPG-galectin binding, emerging from our experimental results.** *Top panel,* modest inflammation caused by the wild type protozoa that results from their good affinity to both galectin-3 and galectin-1, where galectin-3 mediates proinflammatory chemokine production, *e.g.* IL-8, and galectin-1 plays a dual role by suppressing chemokines and stimulating galectin-3 expression. *2nd panel,* higher proinflammatory response caused by the wild type protozoa in the absence of endogenous galectin-1 (achieved in our model by galectin-1 knockdown) and preserved galectin-3 signaling. *3rd panel,* suppressed inflammatory response to the wild type protozoa in the presence of endogenous galectin-1 and absence of endogenous galectin-3 (achieved by galectin-3 knockdown). *4th panel,* suppressed inflammation in response to protozoan mutants that lack affinity to galectin-3 and have reduced but relatively preserved affinity to galectin-1.

In summary, the concept supported by our findings is presented in Fig. 9 and is discussed below.

## Discussion

Since defined 30 years ago, the galectin family has emerged as a key regulator of innate and adaptive immunity (15, 45–47); however, to date very limited information is available on their expression and physiology in the reproductive tract, and in particular, their immune functions in the female genital tract have remained unexplored. Systemic and local levels of galectin-1 have been associated with maternal-fetal tolerance (48). In contrast, cord blood levels of galectin-3 have been positively correlated with common pregnancy complications, *e.g.* preterm delivery and low-for-gestation-age birth weight (49), that have also been significantly associated with maternal *T. vaginalis* infection (2). Galectin-1 and galectin-3 are expressed by virtually all immune cell lineages, including monocytes, macrophages, T cells, NK cells, B cells, and dendritic cells, and have also been implicated in cancer immunology. One study showed galectin expression in mouse vaginal tissue (50). We now demonstrate for the first time expression of galectin-1 and galectin-3 by human epithelial cells originating from all three anatomic compartments of the human female genital tract. We prove their causative roles in the inflammatory reaction elicited by *T. vaginalis*, the most common non-viral sexually transmitted pathogen. Our study also identifies structure-function relationships based on protozoan lipophosphoglycan-galectin affinity underlying *T. vaginalis* signaling to the human host cells. Using mutant parasites with CPI-GC that lacks LacNAc repeats as proven by MS analysis, we demonstrated that the CPI-GC binding to galectin-1 and -3 and its signaling to the host depend on the presence of LacNAc repeats.

Our investigation opened the question of whether extracellular or cell-associated (membrane-bound or intracellular) galectin-1 and -3 mediated the immune responses to *T. vaginalis*. The RNAi approach to knock down both extracellular and intracellular protein expression showed that the reduced protein synthesis led to significant changes in response to *T. vaginalis* infection and that some but not all of these changes were mitigated by adding recombinant galectins. Being synthesized as cytosolic proteins, previous work suggests roles of both intracellular and extracellular galectins in the modulation of immune responses as a part of host-pathogen interactions (15, 46, 47, 51, 52). Importantly, the majority of the work on galectins suggests that extracellular galectins, which are released from cells, are involved in cytokine responses and adhesion of microorganisms to host cells through their binding to glycan ligands. Recent work suggests that cytosolic galectin-3 is concentrated on the membrane of “damaged” lysosomes, from which phagocytosed microorganisms escape into the cytosol, although the physiological significance of the concentration of galectin-3 remains elusive (53, 54). In contrast, some reports suggest the role of intracellular (cytosolic) galectin-3 in the process of phagocytosis of some microorganisms (but not others, such as *Leishmania* and *Streptococcus pneumoniae*), and of galectin-8 (but not galectin-1 or -3) in the initiation of autophagy (21, 22, 55, 56, 58). Although further study would be necessary to address the different roles of other galectins, and the role of their cytosolic compartmentalization in infection with the extracellular parasite *T. vaginalis*, our experiments using recombinant galectin-1 and -3 suggest a significant role of their extracellular levels in *Trichomonas*-host cell interaction.

The experimental results presented here prove that *T. vaginalis* alters directly the extracellular levels of galectin 1- and -3, as well as the amount of these galectins captured by the cervicovaginal epithelial cells, and both recombinant and endogenous galectin-1 and -3, released from the epithelial cells, were decreased in the cell-free medium, especially the levels of galectin-3. Galectin-1 was more abundantly expressed and released at 10-fold higher levels from the cervicovaginal epithelium than galectin-3 and therefore was proportionally less affected by *T. vaginalis* infection. Thus, galectin-3 was more easily depleted when the cells were exposed to parasites with strong affinity to galectin-3.

When epithelial cells were incubated with purified LPG and recombinant galectins, galectin-1 cell-associated levels (measured in cell lysates) increased, although galectin-3 levels did not. This might be due to capturing of the galectin-1·LPG complex at the epithelial cell surface and by oligomerization of galectin-3 within the LPG·glycan complex thus preventing galectin-3 from internalization by the epithelial cells. In support of this interpretation is also the fact that recombinant galectins when mixed with LPG restored the reduced cell-associated levels of galectin-1 but not of galectin-3 in the respective galectin-knockdown phenotypes.

It is also possible that the live parasite has multiple ways of controlling the host environment in addition to the absorption of galectins on its surface and that LPG (CPI-GC) is just one of its virulence factors contributing to the regulation of the cervicovaginal galectin levels. Our results showed that direct contact with the live parasite, to a significantly larger extent than the cell-free fraction of the *Trichomonas* cultures, caused the reduced levels of both galectins. However, a tendency of reduced galectin-1 and -3 levels was shown when recombinant galectins were incubated in the parasite-conditioned medium, suggesting the possibility that *Trichomonas* may be shedding LPG or that at least part of its effects might be driven by proteolytic cleavage by protozoan enzymes.

Our finding that galectin-1 and galectin-3 played opposing roles in the inflammatory responses to *T. vaginalis* infection, supported by both gain and loss of function experiments, is in line with previous reports on the bidirectional functions of these two galectins. For example, galectin-1 may reduce adaptive immune function by inducing IL-10 while suppressing IFNγ in T cells (19, 59); however, while inhibiting leukocyte trafficking and function (15, 60, 61), it can activate some aspects of neutrophil function depending on the stimulatory context (62). Galectin-3 has many proinflammatory activities (15, 60, 61) but is also capable of down-regulating inflammation such as by blocking LPS-induced proinflammatory cytokines (63). We showed that in the context of the cervicovaginal epithelial cells, galectin-1 plays an immunosuppressive and galectin-3 plays an immunostimulatory role. Knocking down galectin-1 in the cervical epithelial cells significantly increased the baseline production of RANTES and MIP-3α, and knocking down galectin-3 suppressed the endogenous production of IL-8. In the context of *T. vaginalis* infection, galectin-1 inhibited the epithelial immune reaction by recognizing the CPI-GC domain presented by LPG on the *T. vaginalis* surface. This was supported by the fact that LPG increased by more than 2-fold the levels of IL-8 and MIP-3α in the knockdown model as compared with the control epithelial cells. The effects of the galectin-1 knockdown on the LPG signaling were mitigated by adding recombinant galectin-1. In contrast, the galectin-3 knockdown decreased the LPG-stimulated IL-8 levels, and this effect could not be mitigated by adding recombinant galectin-3 possibly because, as we discussed above, LPG via its CPI-GC domain can absorb the recombinant galectin-3 or trigger enzymatic degradation and thus may interfere with its uptake by the epithelial cells and downstream signaling. In contrast to IL-8 and MIP-3α, LPG suppressed RANTES levels both in the control and galectin knockdown cells. The differential effects of galectin-1 and galectin-3 are in line with the different functions of the chemokines induced or suppressed by LPG. IL-8 attracts neutrophils and macrophages thus aiding phagocytosis and neutralization of extracellular protozoan pathogens, while MIP-3α is a chemoattractant for dendritic cells thus aiding antigen presentation. Suppressing both of these chemokines via CPI-GC-galectin-1 signaling may aid the survival of the parasite in the epithelial environment. RANTES is different from IL-8 and MIP-3α in that its proinflammatory activity is inversely correlated with its extracellular levels. At low levels RANTES maintains a monomeric or dimeric form that signals through its chemokine-specific receptors CCR1 and -3–5, thereby leading to recruitment of leukocytes to the site of inflammation, hence acting just like IL-8 and MIP-3α. At high levels RANTES stops acting as a chemokine; it multimerizes and signals through cross-linking of glycosaminoglycans with immunostimulating and pro-apoptotic activity (44) Thus, suppressing RANTES levels independently by both galectin-1 and LPG may again support the parasite survival by adherence and exploitation of live epithelial cells. The pathways responsible for the differential effects of galectin-1 and galectin-3 downstream from *T. vaginalis* CPI-GC recognition remain to be elucidated. In contrast to the prototype galectin, galectin-1, in addition to the C-terminal carbohydrate recognition domain, galectin-3, has an N-terminal proline-rich tandem repeat domain allowing it to self-associate in oligomer-engaging multivalent carbohydrate ligands, which may in part explain differences in host receptor engagement and signaling (39–41).

The knockdown of galectin-1 in our experimental model proved that the *T. vaginalis* parasite uses its binding to galectin-1 to suppress immune responses in the vaginal environment. Even though the host recognizes the *Trichomonas* LPG/CPI-GC and initiates an inflammatory reaction, including the release of galectin-3, which enables proinflammatory recognition, the parasite subverts host immunity by hijacking galectin-1 and reducing levels of chemokines responsible for recruitment of neutrophils and macrophages (IL-8), dendritic cells (MIP-3α), and T cells (RANTES), thus creating a less hostile mucosal environment aiding its survival. The loss of chemokine responses to *T. vaginalis* LPG in galectin-3KD epithelial cells and the significantly reduced levels of IL-8 and MIP-3α in response to mutants with truncated CPI-GC, incapable of engaging galectin-3 signaling, underscore the essential role of galectin-3 in the epithelial response against the parasite. Even though epithelial cells released galectin-3 in response to CPI-GC, within 24 h of infection the parasite significantly depleted the total and intracellular stores of galectin-3 thus evading the protective host inflammatory reaction. This phenomenon was observed in infection with both WT and mutant parasites, suggesting a mechanism independent from CPI-GC binding to galectin-3, because the mutant CPI-GC affinity to galectin-3 was drastically reduced. Galectin-1 knockdown decreased the galectin-3 output by the epithelial cells (Fig. 5*D*), and extracellular recombinant galectin-1 in the absence of *Trichomonas* increased the galectin-3 levels (Fig. 4*A*) suggesting that sequestration of epithelial galectin-1 by CPI-GC binding may contribute to the galectin-3 depletion caused by *T. vaginalis*. Alternative mechanisms to be explored include selective enzymatic degradation by protozoan or by host enzymes induced by *T. vaginalis* or by LPG signaling.

CPI-GC isolated from multiple clinical isolates showed comparable affinities to galectin-1, although affinity to galectin-3 varied between isolates from different patients, suggesting a selective pressure by variable host conditions to be addressed by larger future clinical studies. As shown in Fig. 3, galectin-1 binds to CPI-GC of wild type *Trichomonas* (B7RC2) and its mutants (M-4.12 and M-2E2) with a relatively similar affinity. Galectin-1 preferentially binds to β-galactosides proximal to the non-reducing terminal ends of glycans (24, 57). Consistent with this established preference of galectin-1, all CPI-GCs showed high affinity for a plant lectin, ricin (*Ricinus communis* agglutinin), which also binds to non-reducing terminal end β-galactoside. In contrast, galectin-3 failed to show high affinity for the mutant CPI-GCs. Galectin-3 is known to preferentially bind to β-galactoside residues inside the polylactosamine polymer, although it exhibits weak affinity for simply β-galactoside at the non-reducing terminal. Indeed, the mutant CPI-GCs failed to be recognized by tomato lectin that binds to polyLacNAc residues. Together with the results from the knockdown epithelial cell clones, these structure-affinity relationships suggest that galectin-3, but not galectin-1, is involved in the induction of proinflammatory cytokine release through its binding to the parasites. Our findings of selective recognition by plant lectins among the different clinical isolates tested provides further support for the structural diversity among the CPI-GC domains of different clinical isolates. Our experimental results showed that LPG from *T. vaginalis* isolates with different affinity to galectin-3 and galectin-1 induced different levels of proinflammatory responses, *e.g.* IL-8. Whether this diversity may contribute to symptom disparities in trichomoniasis remains to be determined by clinical studies. It remains to be determined what precise molecular features are responsible for variations in galectin-3 binding or possible preferential binding to galectin-1 *versus* galectin-3.

In conclusion, this study provides experimental proof that the *T. vaginalis* parasite utilizes its LPG/CPI-GC binding to galectin-1 and galectin-3 to subvert host immune responses related to clearance of infection in the cervicovaginal environment as schematically summarized in Fig. 9. According to the proposed disease paradigm, *T. vaginalis* via the high affinity of its LPG binding to galectin-1 uses for its own advantage the natural host immune response that the epithelium puts forward to limit inflammatory damage via galectin-1 signaling. At the same time, by depleting the levels of galectin-3, *T. vaginalis* diminishes the LPG pattern recognition through this galectin, which mediates the normal clearance of infection via chemokine-mediated neutrophil and macrophage recruitment. The result of the dual interaction with galectin-1 and -3 is a subdued mucosal environment, less hostile to the parasite, which is in line with clinical findings of the persistent and often silent/asymptomatic nature of this infection. The diversity of CPI-GC binding to galectin-3 should be further studied as a putative molecular basis for symptom disparity in *T. vaginalis* infection. The universally preserved binding to galectin-1 across *T. vaginalis,* if confirmed for a larger number of clinical isolates, could emerge as an evolutionary tool of protozoan parasites to evade host immunity by suppressing protective immune responses and should be further explored along with galectin-3 as a target of future therapeutic approaches.

## Author Contributions

R. N. F. conceived and directed the experiments, secured funding, wrote the manuscript, and coordinated reviews and the contributions of co-authors. B. N. S. contributed to concept development and provided expertise in glycobiology and trichomonad biochemistry. S. S. provided recombinant galectin-3, critical edits, and expertise in galectin glycobiology. H. S. Y., E. F., S. R., and T. F. conducted the experiments, cell viability assessment, and data analysis. H. D., S. R., and N. B. conducted the immunoassays and data analysis. G. R. H. and B. N. S. purified LPG and CPI-GC and performed the galectin-binding experiments. G. S. P. and S. S. purified recombinant galectins and characterized the monoclonal antibodies against galectin-3. All authors read and agreed with the final manuscript content.

## Footnotes

2 The abbreviations used are:

STI: sexually transmitted infection
CPI-CG: ceramide phosphoinositol glycan core
LPG: lipophosphoglycan
RANTES: regulated on activation normal T cell expressed and secreted
polyLacNAc: poly-*N*-acetyl-lactosamine
MTT: 3-(4,5-dimethylthiazol-2-yl)-2,5-diphenyltetrazolium bromide
ANOVA: analysis of variance
m: mutant
rh: recombinant human
LEA: *L. esculentum* agglutinin.
